# Level-Specific Volumetric BMD Threshold Values for the Prediction of Incident Vertebral Fractures Using Opportunistic QCT: A Case-Control Study

**DOI:** 10.3389/fendo.2022.882163

**Published:** 2022-05-20

**Authors:** Michael Dieckmeyer, Maximilian Thomas Löffler, Malek El Husseini, Anjany Sekuboyina, Bjoern Menze, Nico Sollmann, Maria Wostrack, Claus Zimmer, Thomas Baum, Jan Stefan Kirschke

**Affiliations:** ^1^ Department of Diagnostic and Interventional Neuroradiology, School of Medicine, Klinikum rechts der Isar, Technical University of Munich, Munich, Germany; ^2^ Department of Radiology, University Medical Center, Albert-Ludwigs-University Freiburg, Freiburg, Germany; ^3^ Image-Based Biomedical Modeling, Department of Computer Science, Technical University of Munich, Munich, Germany; ^4^ Department of Quantitative Biomedicine, Faculty of Medicine, University of Zurich, Zurich, Switzerland; ^5^ TUM-Neuroimaging Center, Klinikum rechts der Isar, Technical University of Munich, Munich, Germany; ^6^ Department of Diagnostic and Interventional Radiology, University Hospital Ulm, Ulm, Germany; ^7^ Department of Neurosurgery, School of Medicine, Klinikum rechts der Isar, Technical University of Munich, Munich, Germany

**Keywords:** bone mineral density (BMD), osteoporosis, spinal fracture, multidetector computed tomography (MDCT), threshold value

## Abstract

**Purpose:**

To establish and evaluate the diagnostic accuracy of volumetric bone mineral density (vBMD) threshold values at different spinal levels, derived from opportunistic quantitative computed tomography (QCT), for the prediction of incident vertebral fractures (VF).

**Materials and Methods:**

In this case-control study, 35 incident VF cases (23 women, 12 men; mean age: 67 years) and 70 sex- and age-matched controls were included, based on routine multi detector CT (MDCT) scans of the thoracolumbar spine. Trabecular vBMD was measured from routine baseline CT scans of the thoracolumbar spine using an automated pipeline including vertebral segmentation, asynchronous calibration for HU-to-vBMD conversion, and correction of intravenous contrast medium (https://anduin.bonescreen.de). Threshold values at T1-L5 were calculated for the optimal operating point according to the Youden index and for fixed sensitivities (60 – 85%) in receiver operating characteristic (ROC) curves.

**Results:**

vBMD at each single level of the thoracolumbar spine was significantly associated with incident VFs (odds ratio per SD decrease [OR], 95% confidence interval [CI] at T1-T4: 3.28, 1.66–6.49; at T5-T8: 3.28, 1.72–6.26; at T9-T12: 3.37, 1.78–6.36; and at L1-L4: 3.98, 1.97–8.06), independent of adjustment for age, sex, and prevalent VF. AUC showed no significant difference between vertebral levels and was highest at the thoracolumbar junction (AUC = 0.75, 95%-CI = 0.63 - 0.85 for T11-L2). Optimal threshold values increased from lumbar (L1-L4: 52.0 mg/cm³) to upper thoracic spine (T1-T4: 69.3 mg/cm³). At T11-L2, T12-L3 and L1-L4, a threshold of 80.0 mg/cm³ showed sensitivities of 85 - 88%, and specificities of 41 - 49%. To achieve comparable sensitivity (85%) at more superior spinal levels, resulting thresholds were higher: 114.1 mg/cm³ (T1-T4), 92.0 mg/cm³ (T5-T8), 88.2 mg/cm³ (T9-T12).

**Conclusions:**

At all levels of the thoracolumbar spine, lower vBMD was associated with incident VFs in an elderly, predominantly oncologic patient population. Automated opportunistic osteoporosis screening of vBMD along the entire thoracolumbar spine allows for risk assessment of imminent VFs. We propose level-specific vBMD threshold at the thoracolumbar spine to identify individuals at high fracture risk.

## 1 Introduction

Osteoporosis is a systemic skeletal disorder leading to reduced bone quality and increased fracture risk (1). Vertebral fractures (VFs) are one of the most common and clinically relevant fracture sites, accounting for 16% of reported fragility fractures and 53 – 65% of fracture-related deaths in the EU (2). The resulting morbidity, mortality and socioeconomic burden continue to increase (3, 4). A considerable number of patients does not receive existing treatment strategies (4, 5), although it is well known that early initiation in a maximum number of individuals is crucial for these measures to be effective (6). The detection rate of osteoporosis at an early disease stage, preferably before the first fracture occurrence, is low. This is in part due to the insufficient and declining utilization of the currently recommended bone densitometry technique dual energy X-ray absorptiometry (DXA) (7). Furthermore, DXA has limited prediction accuracy of incident VFs which is only marginally improved by spine trabecular bone score (TBS), a gray-level texture analysis tool providing additional microstructural information of DXA images (8). As a promising alternative, quantitative computed tomography (QCT) can overcome many shortcomings of DXA (9, 10). Unlike DXA, QCT measures volumetric bone mineral density (vBMD) using clinical CT scans. Besides their independence from confounding factors like degenerative changes, the three-dimensional measurements facilitate the differentiation of cortical and trabecular bone, which is metabolically more active and therefore more susceptible to osteoporotic changes (9).

Opportunistic QCT refers to bone quality assessment derived from clinical routine CT scans acquired for other indications. In addition to the ability to discriminate osteoporotic from non-osteoporotic subjects (11, 12), it has shown improved prediction of incident VFs compared to DXA (13, 14), making it the most promising tool for opportunistic osteoporosis screening (15). For better clinical interpretation and usage, threshold values for QCT, including trabecular and integral vBMD as well as finite element analysis (FEA)-based bone strength measurements, have been proposed and investigated with regard to diagnosis of osteoporosis (9, 16) and VF risk (14, 17, 18).

Improvements in CT hardware and software have led to faster examinations with lower radiation dose (19), and scanner availability is increasing worldwide (20). Furthermore, the ongoing demographic transition increases the number of patients suffering from age-related diseases, such as cancer, degenerative spine disease, and chronic back pain (21–23), which require CT examinations covering varying parts of the spine. As a result, the amount of CT scans available for opportunistic osteoporosis screening will continue to rise (24).

In previous studies, risk assessment of future VF has mainly been based on measurements at the lumbar spine (14, 17, 25–27). Prevalent VFs at any location of the thoracolumbar spine were shown to be significantly associated with bone measures of both the thoracic and lumbar spine, as assessed by QCT of the single vertebrae T10 and L3 (28). Only recent studies investigated QCT-related incident VF risk at the thoracic spine. While evidence indicates that CT-based vBMD and strength measures of single thoracic and lumbar vertebrae (T8 and L2) can predict incident VFs equally well and irrespective of fracture location (29), the potential use of opportunistic QCT was expanded to the thoracic region by using cardiac CT scans (18, 30).

However, vertebral level-specific vBMD threshold values for the entire thoracolumbar spine, comparable to thresholds recommended for the lumbar spine for the diagnosis of osteoporosis (31), are still lacking, which limits the quantity of data that can be utilized for opportunistic fracture risk assessment. Therefore, additional studies are needed in order to make more clinical CT scans accessible. In the present study, we investigated the association of opportunistic QCT-derived vBMD measurements at different vertebral levels with the risk of incident osteoporotic VF. Furthermore, our goal was to evaluate the diagnostic accuracy of level-specific vBMD thresholds at the thoracolumbar spine for the prediction of incidental VFs and to propose threshold values to identify individuals at high fracture risk.

## 2 Materials And Methods

### 2.1 Study Population

**Figure 1:**
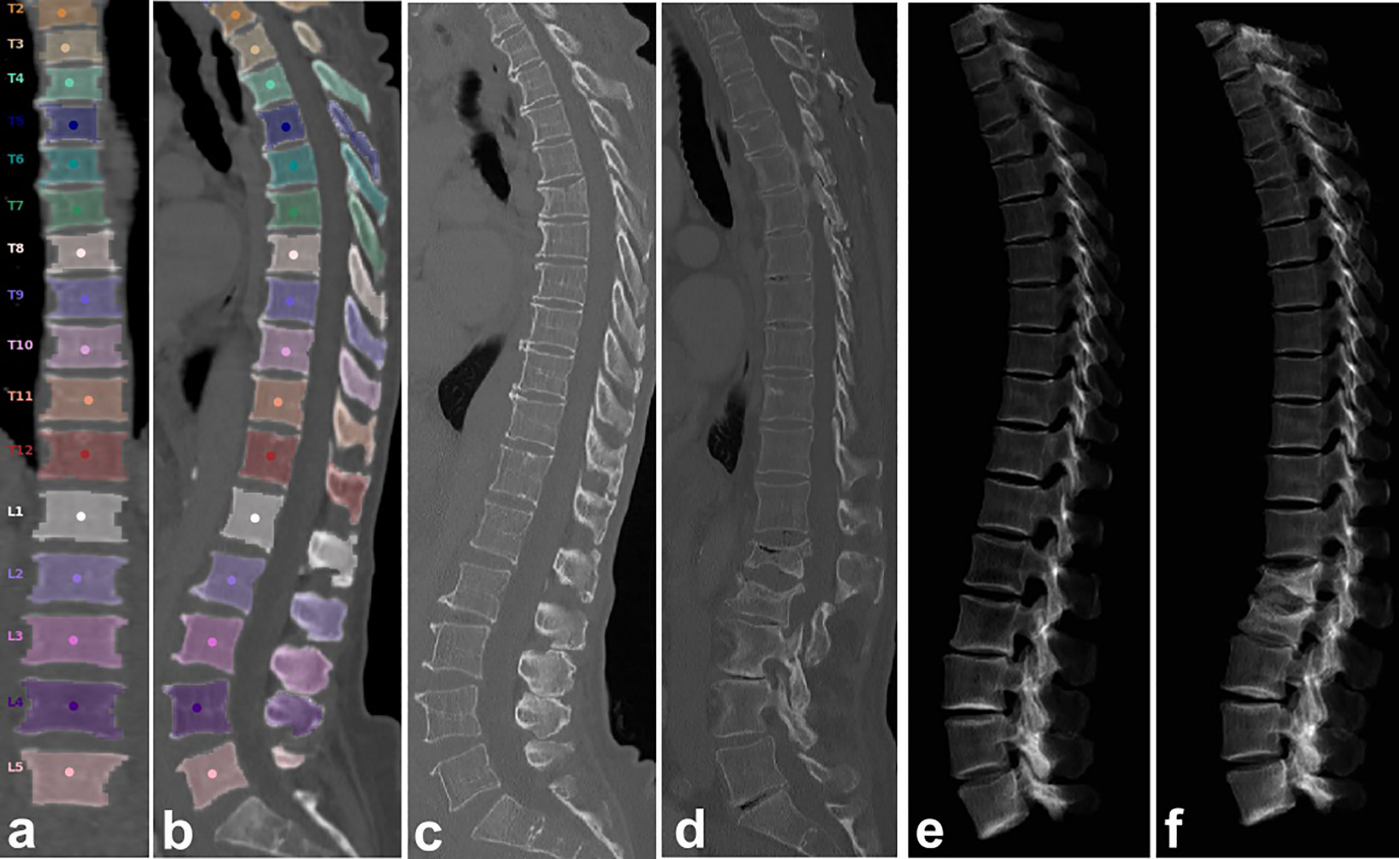
Automated spine labeling and segmentation in coronal **(A)** and sagittal **(B)** view of a baseline CT of a 79-year-old female with trabecular vBMD (L1-L4) = 48.9 mg/cm³. **(C, E)** CT and virtual radiograph at baseline. **(D, F)** CT and virtual radiograph at follow-up after 8 months showing incident vertebral fractures of L1 and L2.

The present case-control study was approved by the local institutional review board. The requirement of written informed consent was waived due to the retrospective nature of the study. In our hospital’s digital picture archiving and communication system (PACS), we searched for multi-detector CT (MDCT) scans covering the thoracolumbar spine that were registered between June 2007 and May 2017. Based on this imaging data, we retrospectively identified patients with and without incident VFs. Exclusion criteria were history of vertebral metastasis or hematologic disorder and CT on a scanner without calibration or with different tube voltage settings. 35 cases with at least one incident VF were identified and included in the study. Patients without incident VFs were matched to cases by sex and age (± 2 years). Among the available matches, two controls per case were selected, prioritizing patients with longer follow-up duration. In total, 70 matched controls were included in the study. Incident VF cases were defined as patients who (i) showed a fractured vertebra in the follow-up scan that was not fred in the baseline scan (**Figure 1**), or (ii) showed an already fractured and consolidated vertebra in the baseline scan that increased by at least one grade on the semi-quantitative Genant scale (32) in the follow-up scan. The absence of bone marrow edema in recent magnetic resonance imaging (MRI) ensured consolidation. Bone marrow edema in MRI or signs of callus formation in CT were interpreted as a sign for active, progressive VF and not considered as incident VF (33, 34).The majority of the study population (91%) received clinical routine CT for oncologic staging, restaging, or follow-up. Oncologic indications included abdominal malignancies (58%), hematological malignancies (18%), renal and urinary tract malignancies (6%), breast cancer (4%), and other malignancies (14%). Other indications included acute or chronic back pain, suspected spinal fracture, postoperative CT after spinal surgery, and exclusion of acute abdominal pathology. Available clinical information relevant to this study can be found in the supplementary material.

### 2.2 CT Imaging

Baseline MDCT examinations were performed on five different scanners in the same hospital (Brilliance 64 and iCT 256, Philips Healthcare; Somatom Definition AS+, Definition AS, and Sensation Cardiac 64, Siemens Healthineers), with or without administration of oral (Barilux Scan, Sanochemia Diagnostics) and intravenous contrast medium (IVCM; Imeron 400, Bracco). Post-contrast scans were acquired in either arterial or portal venous phase, triggered by a threshold of CT attenuation surpassed in a region of interest (ROI) placed in the aorta, or, alternatively, after a delay of 70 s, depending on the clinical indication for CT. Imaging data was acquired in helical mode with a peak tube voltage of 120 kVp, a slice thickness of 0.9 to 1 mm, and adaptive tube load. Sagittal reformations with a slice thickness of 2 or 3 mm were reconstructed using a standard bone kernel and used for VF detection (35).

### 2.3 Opportunistic QCT

#### 2.3.1 Automatic Extraction of vBMD

Volumetric BMD measures were extracted in an automatic multi-step procedure, which required minimal user interaction and was implemented in Python (12). First, vertebrae were automatically segmented in MDCT scans using an automated framework of several consecutive convolutional neural networks (CNNs) that identifies the spine, labels each vertebral body, and creates segmentation masks, individually for each vertebral body (https://anduin.bonescreen.de). Second, single vertebrae were further subsegmented in these masks using affine and deformable transformations to fit templates of vertebral subregions to each vertebral level (12, 36). Among others, the segmented subregions include the trabecular compartment and cortex of the vertebral body, as well as the different parts of the posterior elements. Third, the segmentation masks of the subregions corresponding to the trabecular compartments of the vertebral bodies were used to extract trabecular vBMD.

The vBMD of the trabecular compartment was extracted from the baseline MDCT scans in all available vertebrae from T1 to L5. Vertebra with fractures, severe degenerative changes, hemangiomas or other abnormalities, such as foreign material, leading to alterations of vBMD measurements, were excluded from the analysis. Additionally, measurements were averaged across four consecutive vertebrae and across L1-L2. The rationale was that most single slice QCT protocols include L1 - L3 or L1 - L4, and, to reduce radiation exposure, it is recommended to scan L1- L2 for 3D QCT (9). In case of excluded vertebrae, all available vertebrae of the four levels were considered for the average.

#### 2.3.2 Asynchronous Calibration and Correction for Contrast Agent

CT attenuation is measured in Hounsfield units (HU). We performed HU-to-vBMD conversion using asynchronous calibration based on kVp- and scanner-specific equations (13, 37–39):


$$\text{vBMD}=\mathrm{calibration factor* HU}[\text{mg}/{\mathrm{cm}}^{3}].$$

To obtain the calibration factors, asynchronous phantom measurements with a QRM QSA-717 Phantom (Quality Assurance in Radiology and Medicine GmbH, Germany) with four different hydroxyapatite inserts were performed. The calibration factors for the five utilized scanners and peak tube voltage of 120 kVp are given in **Table 1**. To minimize the measurement error induced by contrast application, automated detection and correction of the presence of contrast agent and the contrast phase was implemented.

**Table 1:** Scanner-specific HU-to-vBMD calibration factors for tube load of 120 kVp.

Manufacturer	Model Name	Calibration factor
PHILIPS	iCT 256	0.72
PHILIPS	Brilliance 64	0.70
SIEMENS	SOMATOM Definition AS	0.71
SIEMENS	SOMATOM Definition AS+	0.72
SIEMENS	Sensation Cardiac 64	0.68

#### 2.3.3 Exclusion of Vertebrae

Curved planar reconstructions (CPRs) in sagittal and coronal view passing through the centroids of vertebral bodies were generated from CT data and overlaid with segmentation masks at 40% opacity (12). Additionally, virtual radiographs in lateral projection were calculated from CT data. Vertebral levels that had to be excluded from the analysis as described above were efficiently identified using these CPRs. If pathologies could not fully be identified or understood in the CPRs, the radiologist had also access to the full 3D MDCT image dataset.

### 2.4 Statistical Analysis

Statistical analysis was performed with SPSS (version 26.0; SPSS Inc., Chicago, IL, USA) and MATLAB (version R2021a, The MathWorks Inc., Natick, MA, USA) using a two-sided level of significance of 0.05 for all statistical tests. Baseline characteristics between patients with and without incident VFs were compared using t-test for continuous variables and Chi-squared test or Fisher’s exact test for categorical variables. Odds ratio (OR) and 95% confidence interval (CI) of incident VFs were calculated using binomial logistic regression models, with unadjusted vBMD, age at baseline, sex, and prevalent VF status at baseline as covariates. OR is expressed per standard deviation (SD) decrease in vBMD for ease of comparability. To evaluate the diagnostic performance for incident VF prediction, receiver operating characteristic (ROC) analysis was performed. Area under the curve (AUC) with 95%-CI was calculated for each single vertebral level as well as combinations of four consecutive vertebral levels, respectively. To provide a reference for comparison to other QCT tools, the described analysis was also performed for the combination of L1 and L2. vBMD threshold values were calculated for the optimal operating point of the ROC curve, according to the Youden index, and a set of fixed minimal sensitivity values, respectively. For comparison of diagnostic performance, sensitivity and specificity were additionally calculated for vBMD = 80 mg/cm³, which is the threshold proposed by the American College of Radiology (ACR) for the diagnosis of osteoporosis at the lumbar spine (31).

## 3 Results

Patients with incident VFs showed no statistically significant differences in sex distribution, age, days to follow-up CT, or the likelihood of prevalent VF in the baseline scan when compared to controls without incident VF (**Table 2**). Trabecular vBMD was significantly lower in the VF cases (p ≤ 0.001). The percentage of patients classified as osteoporotic was significantly higher for the VF cases than for the controls (p ≤ 0.010). For vBMD below a threshold of 80.0 mg/cm³, 85% of the VF cases were classified as osteoporotic at the lumbar spine (L1-L4), compared to 76% at the lower thoracic (T9-T12), 62% at the mid-thoracic (T5-T8), and only 37% at the upper thoracic spine (T1-T4), (**Table 2**).

**Table 2:** Baseline characteristics of patients with and without incident vertebral fracture (VF).

		No incident VF (n = 70)	Incident VF (n = 35)	No incident VF vs.incident VF	All (n = 105)
Females, *n* (%)		46 (66%)	23 (66%)	n.s.	69 (66%)
Age at CT, mean (SD)		66.7 (9.4)	67.4 (11.1)	n.s.	66.9 (10,0)
Days to follow-up CT, median (range)		563 (162–1518)	524 (19-1871)	n.s.	551 (19-1871)
Prevalent fracture, *n* (%)		10 (14%)	16 (46%)	n.s.	26 (25%)
vBMD (SD), T1-T4		118.8 (34.5)	91.8 (32.3)	*p* = 0.001	111.1 (35.9)
Bone density by CT, *n* (%)	Normal	29 (43%)	3 (11%)	*p* = 0.003	32 (34%)
	Osteopenia	29 (43%)	14 (52%)	n.s.	43 (46%)
	Osteoporosis	9 (14%)	10 (37%)	*p* = 0.010	19 (20%)
vBMD (SD), T5-T8		97.4 (32.7)	73.0 (27.2)	*p* = 0.010	90.3 (33.0)
Bone density by CT, *n* (%)	Normal	14 (20%)	3 (10%)	n.s.	17 (17%)
	Osteopenia	33 (47%)	8 (28%)	n.s.	41 (41.5%)
	Osteoporosis	23 (33%)	18 (62%)	*p* = 0.007	41 (41.5%)
vBMD (SD), T9-T12		91.3 (31.3)	68.6 (25.3)	*p* < 0.001	83.9 (31.3)
Bone density by CT, *n* (%)	Normal	14 (20%)	2 (6%)	n.s.	16 (15%)
	Osteopenia	29 (41%)	6 (18%)	*p* = 0.016	35 (34%)
	Osteoporosis	27 (39%)	26 (76%)	*p* < 0.001	53 (51%)
vBMD (SD), L1-L4		83.1 (32.1)	57.9 (26.7)	*p* < 0.001	74.8 (32.5)
Bone density by CT, *n* (%)	Normal	9 (13%)	2 (6%)	n.s.	11 (11%)
	Osteopenia	19 (28%)	3 (9%)	*p* = 0.029	22 (21%)
	Osteoporosis	41 (59%)	29 (85%)	*p* = 0.008	70 (68%)

Bone density was classified as osteopenic for 80.0 mg/cm³ ≤ vBMD < 120.0 mg/cm³, and osteoporotic for vBMD < 80.0 mg/cm³; (n.s., not significant).

The risk of incident VFs was significantly associated with vBMD at the entire thoracolumbar spine, showing an unadjusted OR per SD decrease in vBMD of 3.98 (95%-CI = 1.97 - 8.06) for L1-L4, which remained statistically significant after adjustment for age, sex, and prevalent fracture status (**Table 3**).

**Table 3:** Odds ratios (OR) for the risk of incident VF, unadjusted as well as adjusted for age, sex and prevalent vertebral fracture.

	OR per SD vBMD decrease (95%-CI)			
	Unadjusted	Adjusted for age	Adjusted for age and sex	Adjusted for age, sex and prevalent VF
vBMD T1-T4	3.28 (1.66 – 6.49)	4.85 (2.14 – 10.96)	5.61 (2.32 – 13.57)	5.02 (2.04 – 12.39)
vBMD T5-T8	3.28 (1.72 – 6.26)	4.73 (2.21 – 10.01)	5.12 (2.32 – 11.32)	4.63 (2.02 – 10.62)
vBMD T9-T12	3.37 (1.78 – 6.36)	4.18 (2.07 – 8.45)	4.43 (2.13 – 9.24)	3.69 (1.73 – 7.86)
vBMD L1-L4	3.98 (1.97 – 8.06)	4.98 (2.32 – 10.69)	5.46 (2.40 – 11.95)	4.34 (1.88 – 10.02)
vBMD L1-L2	4.04 (1.94 – 8.42)	5.47 (2.41 – 12.43)	5.97 (2.52 – 14.15)	5.13 (2.07 – 12.68)

**Figure 2:**
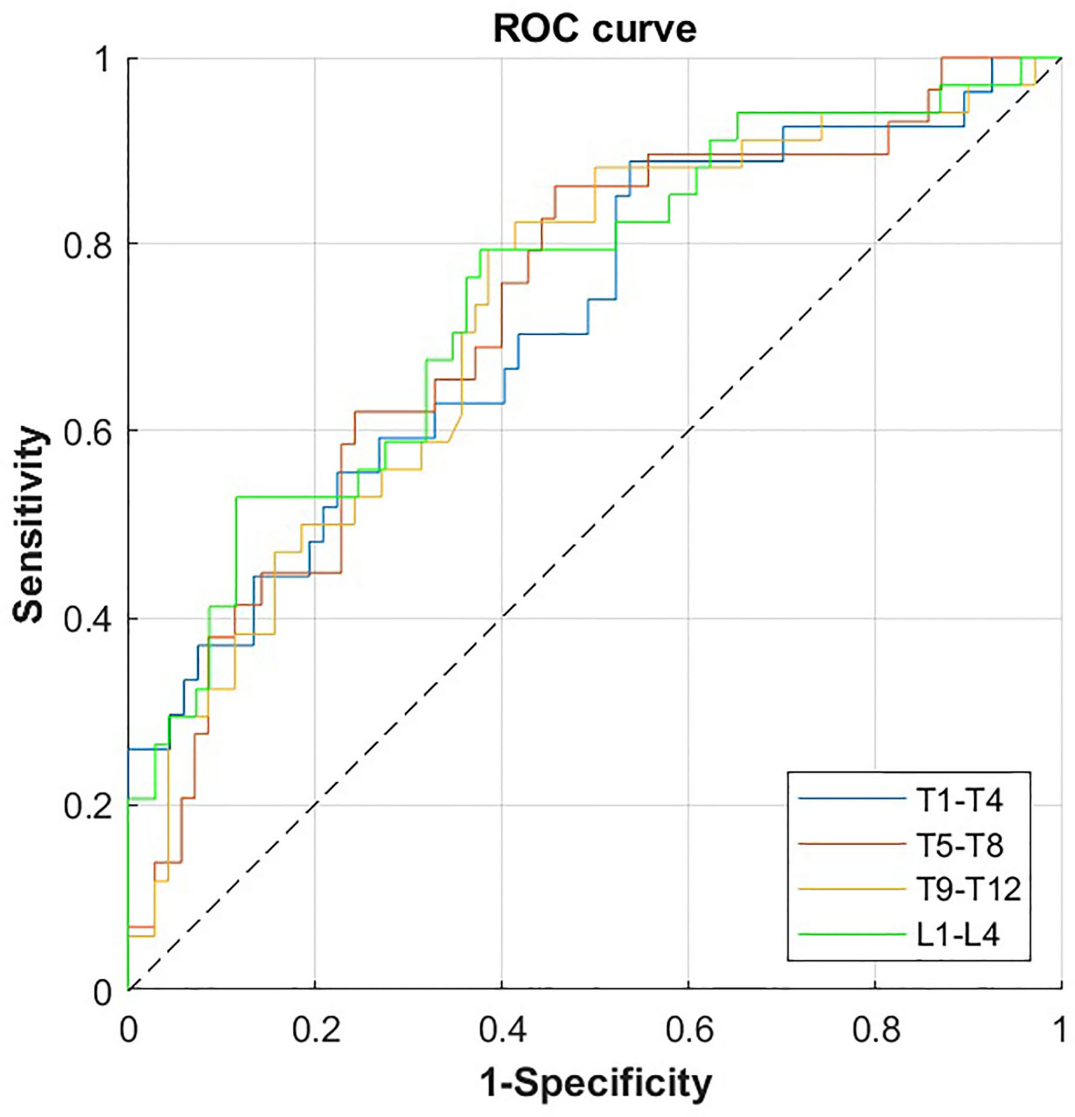
ROC curves for the prediction of incident VF by volumetric bone mineral density (vBMD) for the combination of four consecutive vertebral bodies of the thoracolumbar spine. ROC, receiver operating characteristics; VF, vertebral fracture.

**Figure 3:**
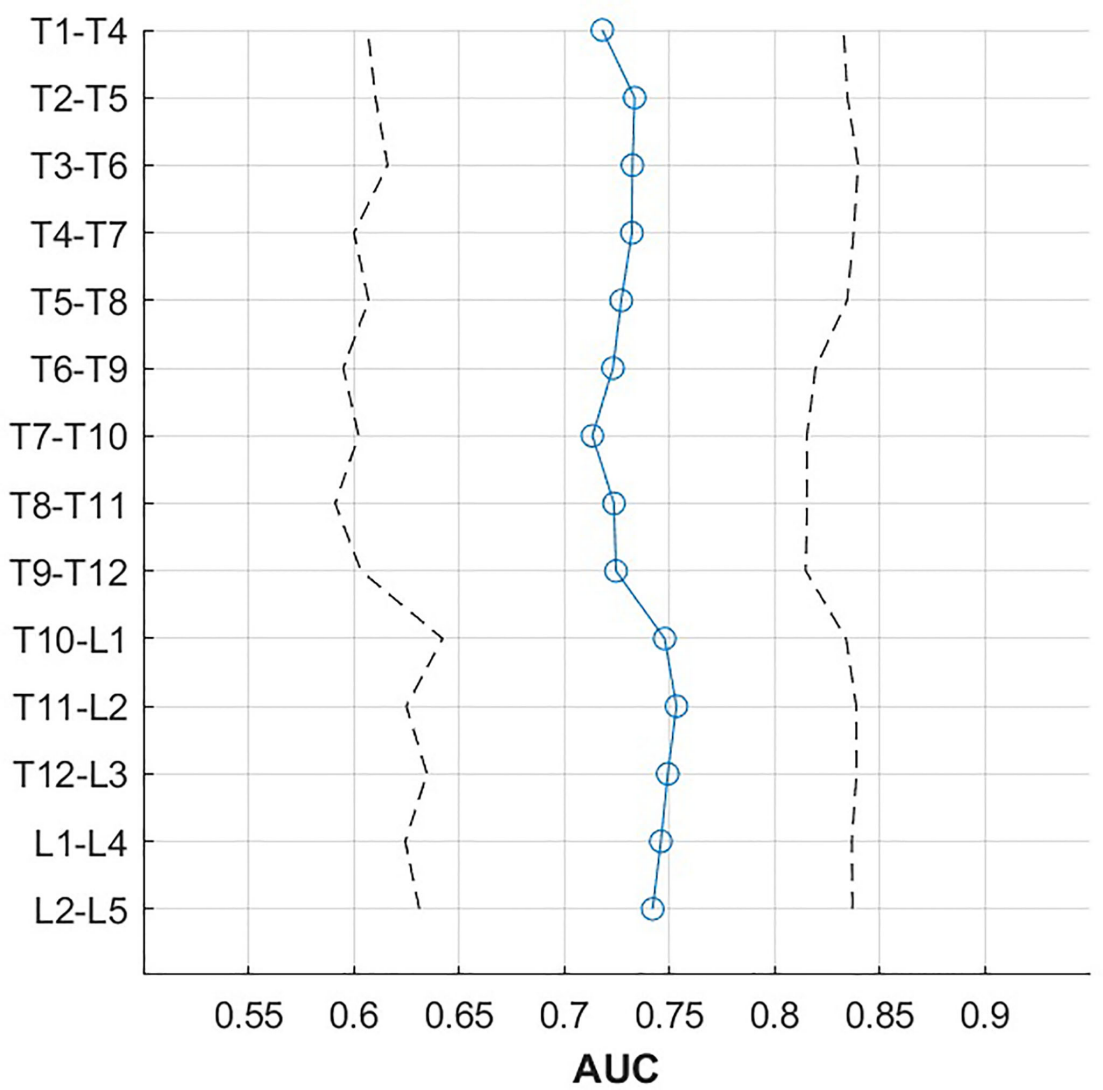
AUC (blue line) with 95% confidence intervals (dashed line) by vertebral height, averaged over four consecutive vertebrae.

In ROC analysis, vBMD was a significant classifier of incident VF status for all single vertebral levels of the thoracolumbar spine (T1 to L5; **Table 4**), as well as for all combinations of four consecutive vertebral bodies (T1-T4 to L2-L5; **Table 5** and **Figure 2**) and L1-L2 (**Table 5**), showing the highest predictive power for T10-L1, T11-L2, T12-L3, L1-L4 and L1-L2 (AUC = 0.75, 95%-CIs = 0.62 - 0.85). Predictive performance showed no significant difference between vertebral levels (p > 0.05; **Table 5** and **Figure 3**).

**Table 4:** Single vertebra analysis: AUC (with 95%-CI), vBMD threshold yielding highest accuracy (TH; mg/cm³), and corresponding sensitivity (SENS) and specificity (SPEC).

Level	AUC	(95%-CI)	TH [mg/cm³]	SENS	SPEC
T1	0.70	(0.56-0.81)	83.1	0.34	0.95
T2	0.71	(0.56-0.82)	71.8	0.30	0.99
T3	0.73	(0.60-0.82)	62.7	0.22	0.99
T4	0.71	(0.60-0.81)	52.6	0.15	1.00
T5	0.73	(0.59-0.84)	66.5	0.50	0.86
T6	0.72	(0.56-0.84)	59.4	0.37	0.92
T7	0.74	(0.61-0.85)	55.9	0.41	0.97
T8	0.72	(0.58-0.83)	42.2	0.12	1.00
T9	0.69	(0.55-0.80)	58.8	0.28	0.93
T10	0.70	(0.58-0.81)	56.1	0.30	0.93
T11	0.72	(0.60-0.81)	52.9	0.35	0.94
T12	0.71	(0.58-0.80)	44.9	0.26	0.96
L1	0.74	(0.60-0.83)	48.5	0.45	0.91
L2	0.73	(0.59-0.82)	46.6	0.46	0.88
L3	0.70	(0.58-0.80)	47.5	0.45	0.89
L4	0.68	(0.56-0.80)	40.9	0.36	0.95
L5	0.71	(0.60-0.82)	48.1	0.28	0.98

**Table 5:** Average across four consecutive vertebrae and L1-L2: AUC (with 95%-CI), optimal vBMD threshold (TH; mg/cm³), and corresponding sensitivity (SENS) and specificity (SPEC).

Level	AUC	(95%-CI)	TH [mg/cm³]	SENS	SPEC
T1-T4	0.72	(0.59-0.82)	69.3	0.26	1.00
T2-T5	0.73	(0.61-0.83)	65.6	0.32	0.97
T3-T6	0.73	(0.59-0.82)	63.1	0.36	0.94
T4-T7	0.73	(0.61-0.83)	59.6	0.36	0.94
T5-T8	0.73	(0.61-0.83)	57.6	0.38	0.91
T6-T9	0.72	(0.58-0.81)	59.5	0.41	0.91
T7-T10	0.71	(0.60-0.82)	61.2	0.39	0.91
T8-T11	0.72	(0.62-0.82)	59.6	0.41	0.91
T9-T12	0.72	(0.61-0.81)	53.6	0.29	0.96
T10-L1	0.75	(0.64-0.84)	51.9	0.34	0.94
T11-L2	0.75	(0.63-0.85)	50.0	0.41	0.93
T12-L3	0.75	(0.62-0.84)	49.6	0.48	0.88
L1-L4	0.75	(0.63-0.84)	52.0	0.53	0.88
L2-L5	0.74	(0.61-0.84)	45.8	0.40	0.93
L1-L2	0.75	(0.63-0.84)	46.9	0.47	0.91

**Figure 4:**
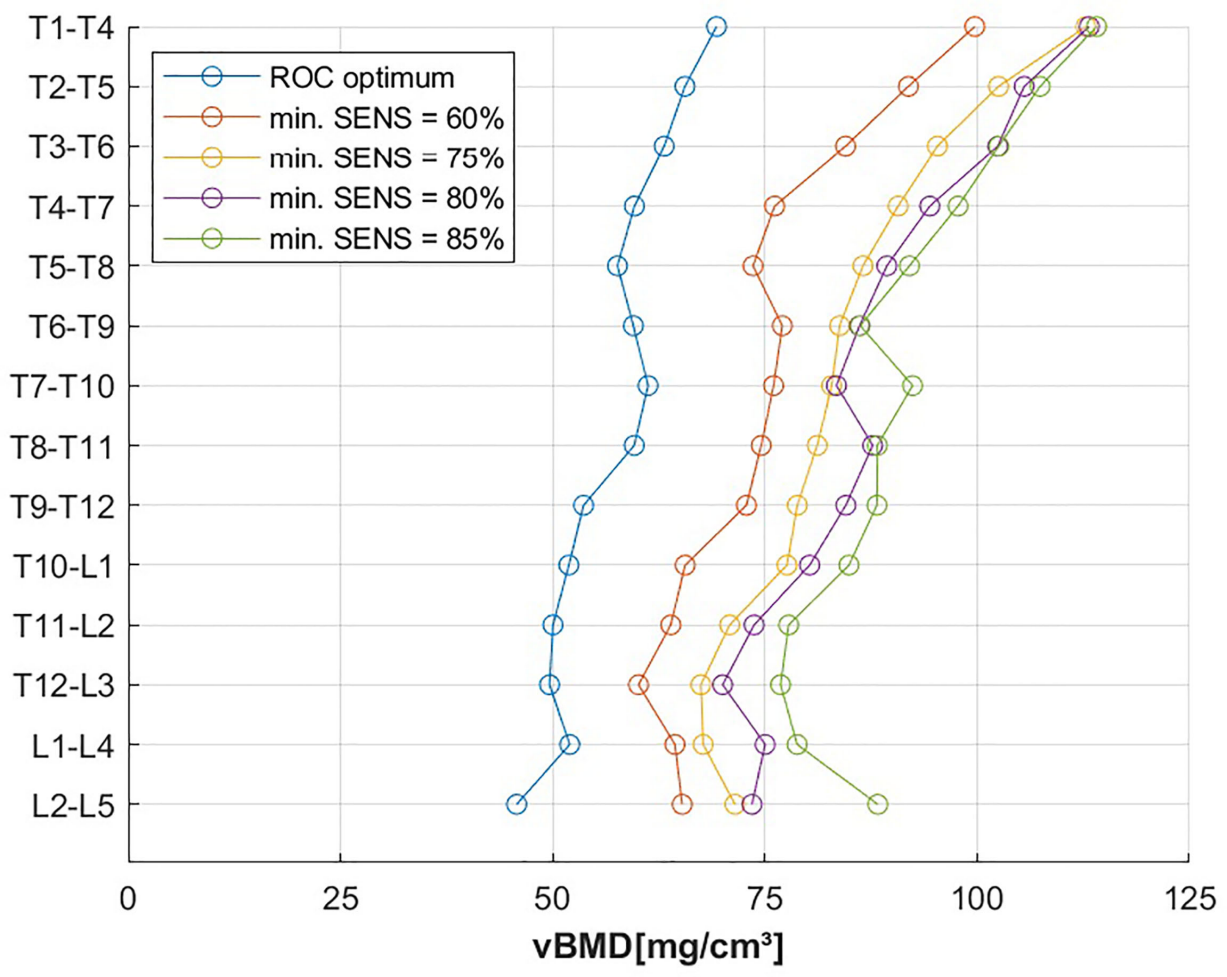
vBMD threshold, grouped by vertebral height for optimal operating point of the ROC curve and fixed minimal sensitivity values (min. SENS).

Vertebral level-specific trabecular vBMD threshold values, as determined by the Youden index of the ROC analysis, ranged from 40.9 to 83.1 mg/cm³ for single vertebral bodies (**Table 4**), and from 45.8 to 69.3 mg/cm³ for the combination of four consecutive vertebral bodies (**Table 5**), showing a tendency to increase from the lumbar to the thoracic spine (**Figure 4**). At the thoracolumbar junction and lumbar spine, a trabecular vBMD threshold value equal to 80.0 mg/cm³ classified incident VFs with a sensitivity between 76% and 88%, and a specificity between 41% and 61% (**Table 6**). Thresholds for the thoracic spine with a fixed minimum sensitivity of 60% were equal to 99.3 mg/cm³ for T1-T4, 73.6 mg/cm³ for T5-8, and 72.8 mg/cm³ for T9-12. For fixed minimum sensitivities of 75%, 80% and 85%, the resulting thresholds were considerably higher, ranging from 67.5 mg/cm³ to 78.8 mg/cm³ at the thoracolumbar junction and lumbar spine, while the corresponding specificities at L1-L4 decreased from 68% to 41% (**Table 6** and **Figure 4**).

**Table 6:** First four columns: vBMD threshold (TH; mg/cm³) for fixed minimal sensitivities (SENS) of 0.60, 0.75, 0.80 and 0.85, along with corresponding specificities (SPEC). Right column: sensitivities and specificities for a fixed vBMD threshold of 80 mg/cm³.

	Min. SENS = 0.60			Min. SENS = 0.75			Min. SENS = 0.80			Min. SENS = 0.85			TH = 80 [mg/cm³]		
Level	TH	SENS	SPEC	TH	SENS	SPEC	TH	SENS	SPEC	TH	SENS	SPEC	TH	SENS	SPEC
T1-T4	99.3	0.63	0.67	112.8	0.78	0.48	113.2	0.81	0.48	114.1	0.85	0.48	80.0	0.37	0.87
T5-T8	73.6	0.62	0.76	86.6	0.76	0.60	89.4	0.83	0.56	92.0	0.86	0.54	80.0	0.62	0.67
T9-T12	72.8	0.62	0.64	78.8	0.76	0.61	84.5	0.82	0.59	88.2	0.85	0.50	80.0	0.76	0.61
T11-L2	63.9	0.62	0.73	70.90	0.76	0.61	73.7	0.82	0.60	77.8	0.85	0.53	80.0	0.85	0.49
T12-L3	60.1	0.61	0.74	67.5	0.76	0.65	70.0	0.82	0.59	76.9	0.88	0.46	80.0	0.88	0.41
L1-L4	64.4	0.62	0.68	67.7	0.76	0.64	75.0	0.82	0.48	78.8	0.85	0.42	80.0	0.85	0.41
L1-L2	62.1	0.63	0.71	65.5	0.75	0.64	67.7	0.81	0.64	77.85	0.88	0.41	80.0	0.88	0.38

## 4 Discussion

In this retrospective case-control study, we derived vertebral level-specific vBMD threshold values associated with an increased risk of incident VFs, using an automated pipeline for opportunistic QCT. Predictive performance was comparable across the entire thoracolumbar spine. Predictive power was highest at the thoracolumbar junction (AUC = 0.75; corresponding sensitivity of 34% to 53% and specificity of 88% to 94% at a threshold of 49.6 to 52.0 mg/cm³), yielding higher AUC values when consecutive vertebral levels were combined. For L1 - L2, we equally found AUC = 0.75. Thus, our results further confirm the official positions of the International Society for Clinical Densitometry (ISCD) which recommend that L1 – L2 should be scanned for 3D QCT (9). Furthermore, lower trabecular vBMD was strongly associated with incident VF risk in a cohort of elderly patients, predominantly examined for oncologic indications.

While there are several longitudinal studies on incident VFs (25–27), comparable studies using opportunistic QCT to assess the association of vBMD and incident VFs are scarce (13, 14, 18, 29). Overall, our results are in accordance with previous findings. Of note, prior studies have evaluated different vertebrae and a variety of quantitative parameters. Trabecular attenuation of L1 derived from opportunistic CT measurements was significantly associated with VF-free survival, however no calibration of the multiple CT scanners was performed (27). In men older than 65 years, integral vBMD of L1, which evaluates the entire vertebral body comprising the trabecular and cortical compartment, showed an AUC of 0.82 for new clinical VFs (25). For the same prospective cohort, trabecular vBMD of L1 to L2 also showed a strong association with future clinical VFs), while predictive performance was comparable (AUC = 0.79 vs. AUC = 0.75) (26). In a comparably designed case-control study investigating the association of incident VF risk and vertebral strength, Allaire et al. reported an AUC of 0.81 for integral vBMD of L3 (14). The mean follow-up time period in this study was 6.0 years, compared to 1.5 years in our study which could explain the higher AUC in their results. In a study population of mainly neurosurgical and oncologic patients, Löffler et al. reported comparable results regarding the association between opportunistic QCT and incident VF risk and equivalent predictive performance (AUC = 0.76) (13). A recent case-control study by Johannesdottir et al. determined CT-based bone measures at two single thoracic and lumbar levels, and demonstrated equivalent ability to predict incident VFs, irrespective of fracture location (29). For vBMD ≤ 80.0 mg/cm³, they reported a sensitivity of 79% and specificity of 47% for L2. At similar spinal levels, our results show equivalent predictive performance, indicated by the comparable sensitivity and specificity of 85% and 41% at L1-4.

In a large prospective cohort study, Therkildsen et al. measured vBMD based on three consecutive thoracic vertebrae in cardiac non-contrast CT examinations to assess the risk of incident VFs which were identified by using patient registries (18). The thresholds established in their study enabled the prediction of incident VFs with moderate performance (AUC = 0.60; sensitivity = 54%, specificity = 66% for vBMD < 102.6 mg/cm³), potentially enabling the use of cardiac scans for opportunistic screening at the thoracic spine. In comparison, we expanded measurements to all thoracolumbar levels and different contrast-enhanced CT scans by using an automated processing pipeline, implementing linear correction equations for the presence of IVCM. In conjunction with the presumably lower number of missed fractures through dedicated imaging follow-up, the level-specific vBMD thresholds we established resulted in a considerably better predictive performance (AUC = 0.73; sensitivity = 62%, specificity = 76% for vBMD < 73.6 mg/cm³ at T5-T8).

We observed a gradient of the derived vBMD thresholds from the lumbar to the thoracic spine. This seems plausible, given that vertebral size decreases in cranial direction and more superiorly located vertebrae tend to be more compact and have higher vBMD per se. Furthermore, cervical and upper thoracic vertebrae are affected less and later by osteoporotic bone mineral loss which potentially contributes to the observed gradient (40). Our data indicates better incident VF prediction for the combination of multiple vertebral levels, which can be considered an extension to previous results by Valentinitsch et al., who demonstrated that all available vertebral levels are important for the best risk assessment of prevalent VFs (41). Furthermore, AUC was highest at the thoracolumbar junction, which we interpret as a result of higher susceptibility and earlier initiation of bone loss in this region (40). We derived vBMD threshold values for the prediction of future VFs by using the optimal operating point of the ROC curve, yielding a value of 64.4 mg/cm³, as well as corresponding sensitivity of 62% and specificity of 68% at L1-L4. Matching the trabecular vBMD threshold proposed by the ACR to be equivalent to the WHO diagnostic threshold for osteoporosis (80.0 mg/cm³) (31) resulted in a sensitivity of 85% and a specificity of 41% (L1-4) in our data. A comparable previous study by Löffler et al. reported a sensitivity of 59% and a specificity of 81% (13). In this study, circular ROIs were manually prescribed within L1 to L4 to extract trabecular vBMD. The differences in sensitivity and specificity could be explained by the different segmentation approach but also by the larger size and different patient selection of our study population.

In clinical practice, not missing a future VF in a patient is arguably more important than the mere accuracy of prediction. Therefore, we additionally calculated thresholds for fixed minimum sensitivities of 60% to 85%, which seem relatively high values, but might reduce the number of false negatives in an average population eligible for opportunistic vBMD screening. However, the currently recommended threshold of 80 mg/cm^3^ yields a rather low specificity, which seems clinically acceptable, in particular in an opportunistic screening setting since it could be compensated by subsequent clinical fracture risk assessment (42). The low specificity in our data may also be due to the limited follow-up time period. The differences found between the level-specific thresholds should be considered when used for interventional decisions, and threshold values should potentially be applied on a patient-specific basis.

We used asynchronous calibration with tube voltage- and scanner-specific HU-to-vBMD conversion equations. These conversion equations were developed based on previous results demonstrating high correlation coefficients (37–39). In comparison to conventional QCT with synchronous calibration, asynchronous QCT has shown good short-term precision and low precision errors of intra-observer and inter-observer reproducibility before (43, 44). To further increase the comparability of the extracted values, an automated CNN-based detection and correction was applied to account for vBMD bias related to contrast agent application (45, 46). The automated labeling and segmentation framework implies that minimum user interaction is required for opportunistic QCT-based vBMD extraction, enabling a time- and cost-efficient clinical implementation of an opportunistic risk assessment for future VFs.

The following limitations of this retrospective case-control study have to be acknowledged. First, the number of VF cases was relatively low and the study population exhibits a bias towards elderly oncologic patients with Caucasian descent. This might restrict the generalizability of our findings, and thresholds might not be easily transferred to other patient groups. However, age, oncologic disease and related treatment are major risk factors, and these patient groups would particularly benefit from opportunistic VF prediction. Second, risk assessment was only performed on a subject level, not predicting the site of fracture. However, such information can be considered of minor relevance, since clinical consequences are mostly independent of the exact level of a VF. Furthermore, it is very difficult to get a sufficient amount of data to perform a valid analysis of site-specific incident VF predictions. Third, the median follow-up duration of 1.5 years was rather short in our study. For example, clinical fracture risk is usually calculated based on a 10-year time period (42). Since a shorter follow-up duration implicates higher specificity and lower sensitivity, this might reduce the comparability to other studies. Forth, the prevalence of oncologic disease was different between cases and controls which could have a biasing effect. It has to be acknowledged that due to limited access to patient records and incompletely documented medical history, no perfectly reliable patient information on relevant pharmacotherapy, chemotherapy, radiation therapy and comorbidities could be obtained. However, overall available clinical information suggests that the prevalence of oncologic disease is the only potential confounder.

## 5 Conclusion

In an elderly, predominantly oncologic patient cohort, lower vBMD from opportunistic QCT of the thoracolumbar spine was associated with increased risk of incident VF. An automated opportunistic screening enables the reliable prediction of future VFs, can be based on clinical routine CT examinations along the entire thoracolumbar spine and performed with different scanners and in different contrast media phases. Desired sensitivity and location of the analyzed spine region should be taken into account for the clinical application of level-specific vBMD threshold values.

## Data Availability Statement

The raw data supporting the conclusions of this article will be made available by the authors, without undue reservation.

## Ethics Statement

The studies involving human participants were reviewed and approved by Ethikkommission der Technischen Universität München. Written informed consent for participation was not required for this study in accordance with the national legislation and the institutional requirements.

## Author Contributions

Authors have made the following contributions. Conceptualization: MD, ML, TB, and JK. Methodology: MD, ML, AS, BM, and JK. Formal analysis: MD, MS, ML, and JK. Investigation: MD, ML, NS, TB, and JK. Resources: BM, CZ, TB, and JK. Data curation: MD, ML, ME, AS, MW, and JK. Writing—original draft preparation: MD and JK. Writing—review and editing: MD, ML, NS, TB, and JK. Visualization: MD, ML, and ME. Supervision: TB and JK Project administration: JK and CZ. Funding acquisition: TB and JK. All authors have read and agreed to the published version of the manuscript.

## Funding

JK and TB have received funding by the German Research Foundation (Deutsche Forschungsgemeinschaft, DFG; project 432290010). JK has received funding by European Research Council (ERC) under the European Union’s Horizon 2020 research and innovation programme (grant agreement No 963904 – Bonescreen – ERC-2020-POC-LS).

## Conflict of Interest

The authors declare that the research was conducted in the absence of any commercial or financial relationships that could be construed as a potential conflict of interest.

## Publisher’s Note

All claims expressed in this article are solely those of the authors and do not necessarily represent those of their affiliated organizations, or those of the publisher, the editors and the reviewers. Any product that may be evaluated in this article, or claim that may be made by its manufacturer, is not guaranteed or endorsed by the publisher.

## Abbreviations

ACR, American College of Radiology; AUC, Area under the curve; BMD, Bone mineral density; CI, Confidence interval; CNN, Convolutional neural network; CPR, Curved planar reconstruction; CT, Computed tomography; DXA, Dual-energy X-ray absorptiometry; EU, European union; FEA, Finite element analysis; HU, Hounsfield units; ISCD, International Society of Clinical Densitometry; IVCM, Intravenous contrast medium; MDCT, Multi-detector computed tomography; MRI, Magnetic resonance imaging; OR, Odds ratio; PACS, Picture archiving and communication system; QCT, Quantitative computed tomography; ROC, Receiver operating characteristic; ROI, Region of interest; SD, Standard deviation; SENS, Sensitivity; SPEC, Specificity; TH, vBMD threshold; vBMD, Volumetric bone mineral density; VF, fracture; WHO, World Health Organization.
