# Supplementary material for: Level-Specific Volumetric BMD Threshold Values for the Prediction of Incident Vertebral Fractures Using Opportunistic QCT: A Case-Control Study
Source: Front Endocrinol (Lausanne). 2022 May 20;13:882163. doi: 10.3389/fendo.2022.882163 (PMC9165054; doi:10.3389/fendo.2022.882163)
Supplement: Supplementary file 1 [file DataSheet_1.pdf]

Supplementary material

	No incident VF (n=70)	Incident VF (n=35)	No incident VF vs. incident VF	All (n=105)
Oncologic disease, *n* (%)	70 (100%)	26 (74%)	$p < 0.001$	96 (91%)
Vitamin D therapy, *n* (%)	2 (3%)	7 (20%)	$p = 0.006$	9 (9%)
Antiresorptive therapy, *n* (%)	0 (0%)	3 (9%)	$p = 0.035$	3 (3%)
Chemotherapy, *n* (%)	50 (71%)	19 (54%)	n.s.	69 (66%)
Radiotherapy, *n* (%)	16 (23%)	8 (23%)	n.s.	24 (23%)
Glucocorticoid treatment, *n* (%)	10 (14%)	6 (17%)	n.s.	16 (15%)
Diabetes, *n* (%)	8 (11%)	7 (20%)	n.s.	15 (14%)

Table S1: Clinical information of patients with and without incident vertebral fracture (VF). (n.s., not significant). Note: Due to heterogeneous and imperfectly documented patient records, data on pharmacotherapy (Vitamin D, antiresorptive therapy, glucocorticoids) and type 2 diabetes has to be considered with caution.

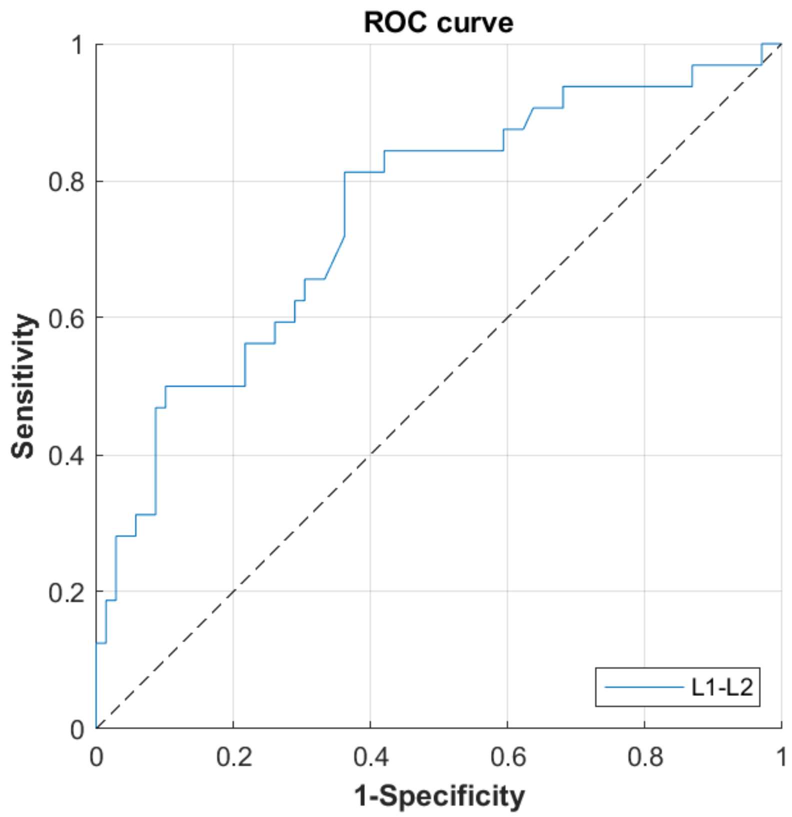

Figure S1: ROC curve for the prediction of incident VF by volumetric bone mineral density (vBMD) for L1 – L2. ROC, receiver operating characteristics; VF, vertebral fracture.
